# Pinocembrin ameliorates atrial fibrillation susceptibility in rats with anxiety disorder induced by empty bottle stimulation

**DOI:** 10.3389/fphar.2022.1004888

**Published:** 2022-10-20

**Authors:** Qian Ran, Cui Zhang, Weiguo Wan, Tianxin Ye, Ying Zou, Zhangchi Liu, Yi Yu, Junhua Zhang, Bo Shen, Bo Yang

**Affiliations:** ^1^ Department of Cardiology, Renmin Hospital of Wuhan University, Wuhan, China; ^2^ Cardiovascular Research Institute, Wuhan University, Wuhan, China; ^3^ Hubei Key Laboratory of Cardiology, Wuhan, China; ^4^ Humanwell Healthcare, Wuhan, China

**Keywords:** pinocembrin, anxiety disorder, atrial fibrillation, electrophysiological remodeling, oxidative stress

## Abstract

**Background:** Anxiety disorder (AD) is the most common mental disorder, which is closely related to atrial fibrillation (AF) and is considered to be a trigger of AF. Pinocembrin has been demonstrated to perform a variety of neurological and cardiac protective effects through its anti-inflammatory and antioxidant activities. The current research aims to explore the antiarrhythmic effect of pinocembrin in anxiety disorder rats and its underlying mechanisms.

**Methods:** 60 male Sprague-Dawley rats were distributed into four groups: CTL group: control rats + saline; CTP group: control rats + pinocembrin; Anxiety disorder group: anxiety disorder rats + saline; ADP group: anxiety disorder rats + pinocembrin. Empty bottle stimulation was conducted to induce anxiety disorder in rats for 3 weeks, and pinocembrin was injected through the tail vein for the last 2 weeks. Behavioral measurements, *in vitro* electrophysiological studies, biochemical assays, ELISA, Western blot and histological studies were performed to assess the efficacy of pinocembrin. In addition, HL-1 atrial cells were cultured *in vitro* to further verify the potential mechanism of pinocembrin.

**Results:** After 3 weeks of empty bottle stimulation, pinocembrin significantly improved the exploration behaviors in anxiety disorder rats. Pinocembrin alleviated electrophysiological remodeling in anxiety disorder rats, including shortening the action potential duration (APD), prolonging the effective refractory period (ERP), increasing the expression of Kv1.5, Kv4.2 and Kv4.3, decreasing the expression of Cav1.2, and ultimately reducing the AF susceptibility. These effects may be attributed to the amelioration of autonomic remodeling and structural remodeling by pinocembrin, as well as the inhibition of oxidative stress with upregulation of the nuclear factor erythroid 2-related factor 2 (Nrf2) and heme oxygenase-1 (HO-1) pathway.

**Conclusion:** Pinocembrin can reduce AF susceptibility in anxiety disorder rats induced by empty bottle stimulation, with the inhibition of autonomic remodeling, structural remodeling, and oxidative stress. Therefore, pinocembrin is a promising treatment for AF in patients with anxiety disorder.

## 1 Introduction

Atrial fibrillation (AF) is the most common persistent arrhythmia clinically, involving 2%–4% adults with an estimated 2.3-fold increase due to longevity (Hindricks et al., 2020). AF is often associated with multiple diseases, including heart failure, valvular diseases, cardiomyopathy, and psychiatric disorders, resulting in an increased mortality due to the high incidence of thromboembolism (Lip et al., 2016). Therefore, it is necessary to explore safe and effective preventions and treatments of AF due to the huge economic and mental burden it brings to patients.

Anxiety disorder (AD) is a mixed feeling of restlessness, fear and worry, accompanied by accelerated heart rate and sleep disturbances (Pitsavos et al., 2006). AD is the most common mental disorder, affecting 33.7% of the population, but rarely receives attention from psychiatrists (Bandelow and Michaelis, 2015). Pervious researches have demonstrated that AD increased the incidence of postoperative AF and affected the symptom severity and treatment success rate of AF (Tully et al., 2011; Thompson et al., 2013). AD is considered to be a possible trigger of AF, in which inflammation and oxidative stress play a key role (Severino et al., 2019).

Pinocembrin is a flavonoid extracted from propolis and other plants, and has demonstrated a variety of neurological and cardiac protective effects through its anti-inflammatory and antioxidant activities (Rasul et al., 2013). For instance, pinocembrin ameliorated depression-like behavior and neuronal apoptosis by inhibiting oxidative stress in a mouse model of depression (Wang et al., 2020), and ameliorated cognitive impairment and neurovascular damage in mice with Alzheimer’s disease (Liu et al., 2014). In addition, pinocembrin attenuated myocardial injury and cardiac dysfunction in sepsis rats and heart failure rats by suppressing inflammation and oxidative stress, respectively (Chen et al., 2021; Li et al., 2021). Our previous study has exhibited that pinocembrin relieved AF and ventricular fibrillation (VF) in depression rats (Ye et al., 2020).

Based on the dual neural and cardiac protective effects of pinocembrin, we hypothesized that pinocembrin could ameliorate atrial fibrillation in AD rats. Therefore, the study was designed to explore the effect of pinocembrin on AF in AD rats and explore the potential mechanism.

## 2 Materials and methods

### 2.1 Materials and experimental protocol

60 male Sprague-Dawley rats (200 ± 20 g) provided by the Experimental Animal Center of Wuhan University were raised in the barrier environment with free availability of water and food (Animal Ethical Number: 20211003). After 1 week of adaptation, SD rats were randomly distributed into four groups: CTL group: control rats + saline; CTP group: control rats + pinocembrin; AD group: anxiety disorder rats + saline; ADP group: anxiety disorder rats + pinocembrin.

Empty bottle stimulation is a well-established method to induce anxiety disorder in rats (Feng et al., 2018). Briefly, rats in the four groups were allowed to drink water only at 9:00–9:10 and 21:00–21:10 each day for the first week to adjust to the new drinking habits. For the next 2 weeks, rats in the AD group and ADP group were randomly given empty bottles once a day at the two designated times.

Pinocembrin (5 mg/kg) was injected into tail vein in the CTP rats and the ADP rats in the last 2 weeks, and saline was injected into the CTL rats and the AD rats.

### 2.2 Open field test

After 3 weeks of empty bottle stimulation, the open field experiment was performed. The rats were placed in the center of the open field, which was divided into nine grids on average, for a total of 5 min. The number of central entries, the cumulative time in center, and the number of rearing were recorded.

### 2.3 Heart rate variability (HRV) analysis

A 15-min surface electrocardiogram from lead II was recorded from rats anesthetized with pentobarbital sodium (60 mg/kg). The surface electrocardiogram was analyzed by LabChart 8.0 software to obtain heart rate variability, which included time domain parameters (average of RR intervals (average RR), standard deviation of RR intervals (SDRR), and square root of the mean squared differences of successive RR intervals (RMSSD)) and frequency domain parameters (low frequency from 0.2 to 0.75 Hz (LF), high frequency from 0.75 to 2.50 Hz (HF), and the ratio of LF to HF).

### 2.4 *In vitro* electrophysiology analysis

#### 2.4.1 Heart isolation

The rats were anesthetized 15 min after peritoneal injection of heparin sodium (400 units), then the hearts were removed and immediately connected with the Langerdorff system, which were perfused with HEPES-buffered Tyrode solution (Liu et al., 2018).

#### 2.4.2 Recording of monophasic action potentials

The paired platinum-stimulating electrodes (diameter: 0.25 mm; spacing: 1 mm) were placed in the left atrial appendage for programmed stimulation, and the custom-made Ag–AgCl electrodes (diameter: 0.25 mm; spacing: 0.5 mm) were placed in the left atrial appendage 1 cm away from the paired platinum-stimulating electrodes for recording monophasic action potentials (MAPs).

#### 2.4.3 Action potential duration analysis

Action potential duration (APD) was obtained by the S1S1 stimulation procedure, which consisted of 10 stimuli at pacing cycle lengths (PCLs) of 250 ms, 200 ms, 150 ms, and 100 ms. The duration of the action potential repolarized to 90% and 50% were recorded.

#### 2.4.4 Effective refractory period analysis

Effective refractory period (ERP) was stimulated by the S1S2 procedure, which consisted of eight successive stimuli (S1) (cycle length (CL): 200 ms) followed by a preceding stimulus (S2). The interval of S2 was reduced from 100 ms to 1 ms, and the longest S2 interval without capturing the atrium was defined as ERP.

#### 2.4.5Atrial fibrillation (AF) inducibility

A 50-Hz burst pacing procedure within 6 times was conducted to induce AF, which was defined as rapid irregular atrial rhythm occurring and maintaining for at least 2 seconds (Liu et al., 2018; Ye et al., 2019).

### 2.5 Histological analysis

Left atrial tissues were embedded in paraffin and sliced into 5um sections, which were incubated with anti-tyrosine hydroxylase (TH) antibody and anti-growth-associated protein-43 (GAP43) antibody for immunostaining, and Sirius red staining was performed to measure atrial fibrosis.

### 2.6 HL-1 cells culture and treatment

To further verify the potential mechanisms of pinocembrin, HL-1 cells derived from mice heart atrium were cultured *in vitro*. Hl-1 cells were cultured in Dulbecco’s modified Eagle’s medium (DMEM)/F12 medium containing 10% fetal bovine serum and 1% penicillin/streptomycin in an incubator containing 5% carbon dioxide at 37°C. The cells were distributed into six groups: (i) CTL group: normal HL-1 cells; (ii) CTL + Pino group: normal HL-1 cells administered with pinocembrin; (iii) CTL + Pino + ML385: normal HL-1 cells administered with pinocembrin and ML385; (iv)H2O2 group: HL-1 cells administered with H2O2; (v) H2O2+Pino group: HL-1 cells administered with pinocembrin and H2O2; (vi) H2O2+Pino + ML385 group: HL-1 cells administered with pinocembrin, ML385 and H2O2. Pinocembrin (25 μM) and ML385 (Nrf2 inhibitor, 5 μM) were added into the medium for 4 h, followed by H2O2 (200 μM) for 3 h to induce oxidative stress.

### 2.7 ROS measurement

Frozen left atriums and cell climbing slices were stained with 5 mM DHE at 37°C for 30 min and then observed by fluorescence microscopy to evaluate the fluorescence intensity of ROS.

### 2.8 ELISA

Inferior venous blood, left atrium and culture supernate were extracted for ELISA and biochemical detection. The blood was centrifuged at 3000 g for 15 min at 4°C to separate the serum. The concentration of norepinephrine (NE) was measured in serum and left atrium.

### 2.9 Biochemical detection

Biochemical detection was performed to assess the concentration of MDA and the activity of SOD in serum, left atrium, and culture supernate.

### 2.10 Western blot analysis

Tissues from left atrium were used for western blot, and proteins including Nrf2 (1:1,000; Abcam), HO-1 (1:1,000; Abcam), Cav1.2 (1:1,000, Abcam), Kv1.5 (1:1000, Bioss), Kv4.2 (1:1000, Abcam), Kv4.3 (1:1000, Bioss), Cx40 (1:1000, Bioss), TH (1:1000, Abcam), GAP43 (1:1000, Abcam), TGF-β1 (1:1000, Abcam), Collagen I (1:1000, Bioss), Collagen III (1:1000, Bioss) and GAPDH (1:1000, Servicebio) were measured based on our previous study (Chen et al., 2020).

### 2.11 Statistical analysis

Continuous variables were presented as means ± standard errors, and categorical variables were presented as counts and percentages (%). Comparisons of continuous variables between two groups were conducted by Student’s t-test, and were conducted by analysis of variance (ANOVA) between multiple groups appropriately. And categorical variables were calculated by the contingency table Pearson’s chi-squared-test or Fisher exact calculation. *p* < 0.05 was defined statistically significant.

## 3 Results

### 3.1 Pinocembrin ameliorated anxiety symptoms induced by empty bottle stimulation

**FIGURE 1:**
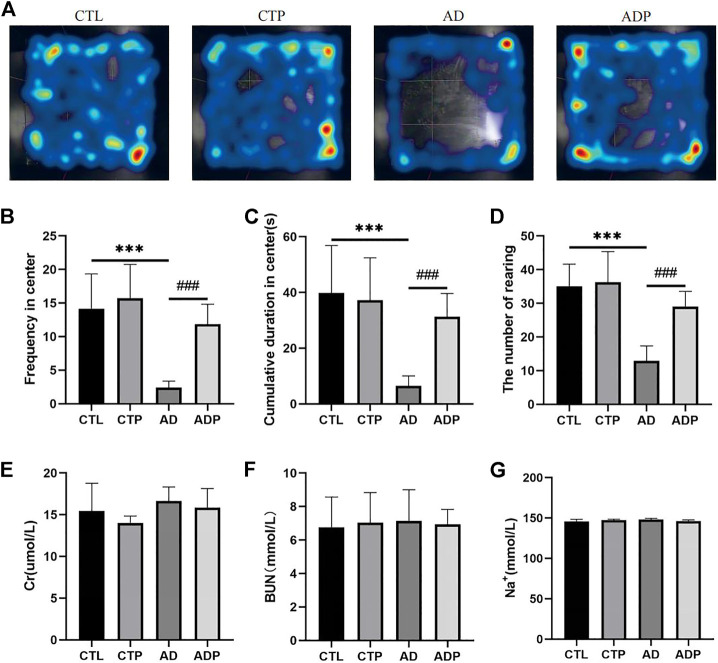
Behavioral measurements. **(A)** Typical images of trajectory after empty bottle stimulation. **(B)** Frequency of central entries. **(C)** Cumulative time in center. **(D)** Number of rearing. n = 15 per group. **(E–G)** The concentration of creatinine (Cr), urea nitrogen (BUN) and Na^+^ in serum. *p < 0.05, **p < 0.01, ***p < 0.001 vs CTL; #*p* < 0.05, ##*p* < 0.01, ###*p* < 0.001 vs. AD.

After the empty bottle stimulation, the frequency of central entries, the cumulative time in center, and the number of rearing were significantly reduced in the AD rats compared with the CTL rats, indicating a weakened exploration behavior in the AD rats (Figures Fig1A–D). However, all the behavioral parameters were significantly improved in the ADP rats compared with the AD rats, and no apparent discrepancy was exhibited between the CTL rats and the CTP rats. In order to exclude the influence of dehydration, the concentration of serum creatinine (Cr), urea nitrogen (BUN) and Na^+^ was measured, and the results showed that there were no significant differences among the four groups (Figures 1E–G).

### 3.2 Pinocembrin restored HRV in anxiety disorder rats

**FIGURE 2:**
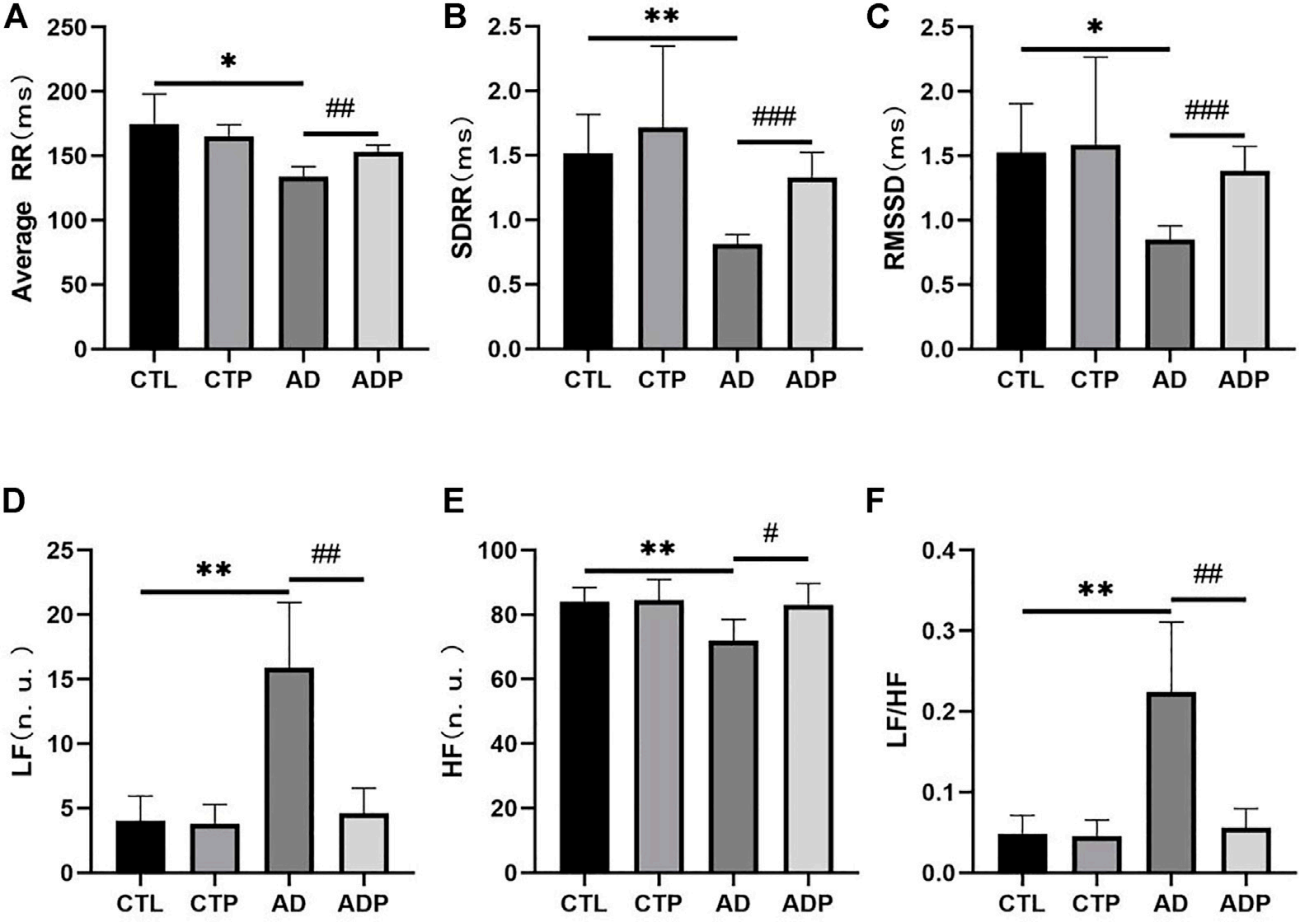
Heart rate variability analysis. **(A–C)** Average RR, SDRR, RMSSD. **(D–F)** LF, HF and LF/HF. *n* = 7 per group. *p < 0.05, **p < 0.01, ***p < 0.001 vs. CTL; #*p* < 0.05, ##*p* < 0.01, ###*p* < 0.001 vs AD.

A significant reduction of time domain parameters (average RR, SDRR, and RMSSD) was exhibited in the AD rats compared with the CTL rats (Figures 2A–C). In respect of frequency domain parameters, LF and LF/HF were obviously higher in the AD rats than the CTL rats, while HF was lower in the AD rats (Figures 2D–F). In addition, there was no obvious difference in the HRV parameters between the CTL rats and the CTP rats.

### 3.3 Pinocembrin ameliorated repolarization characteristics in anxiety disorder rats

**FIGURE 3:**
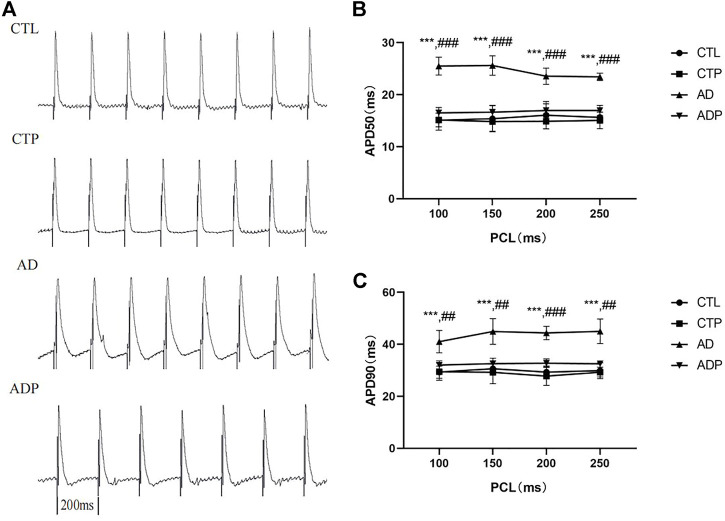
Action potential duration. **(A)** Typical recordings of APD at PCL of 200 ms. **(B,C)** The spatial dispersions of APD_50_ and APD_90_ at PCL of 250 ms, 200 ms, 150 ms, 100 ms. *n* = 7 per group. *p < 0.05, **p < 0.01, ***p < 0.001 vs. CTL; #*p* < 0.05, ##*p* < 0.01, ###*p* < 0.001 vs. AD.

**FIGURE 4:**
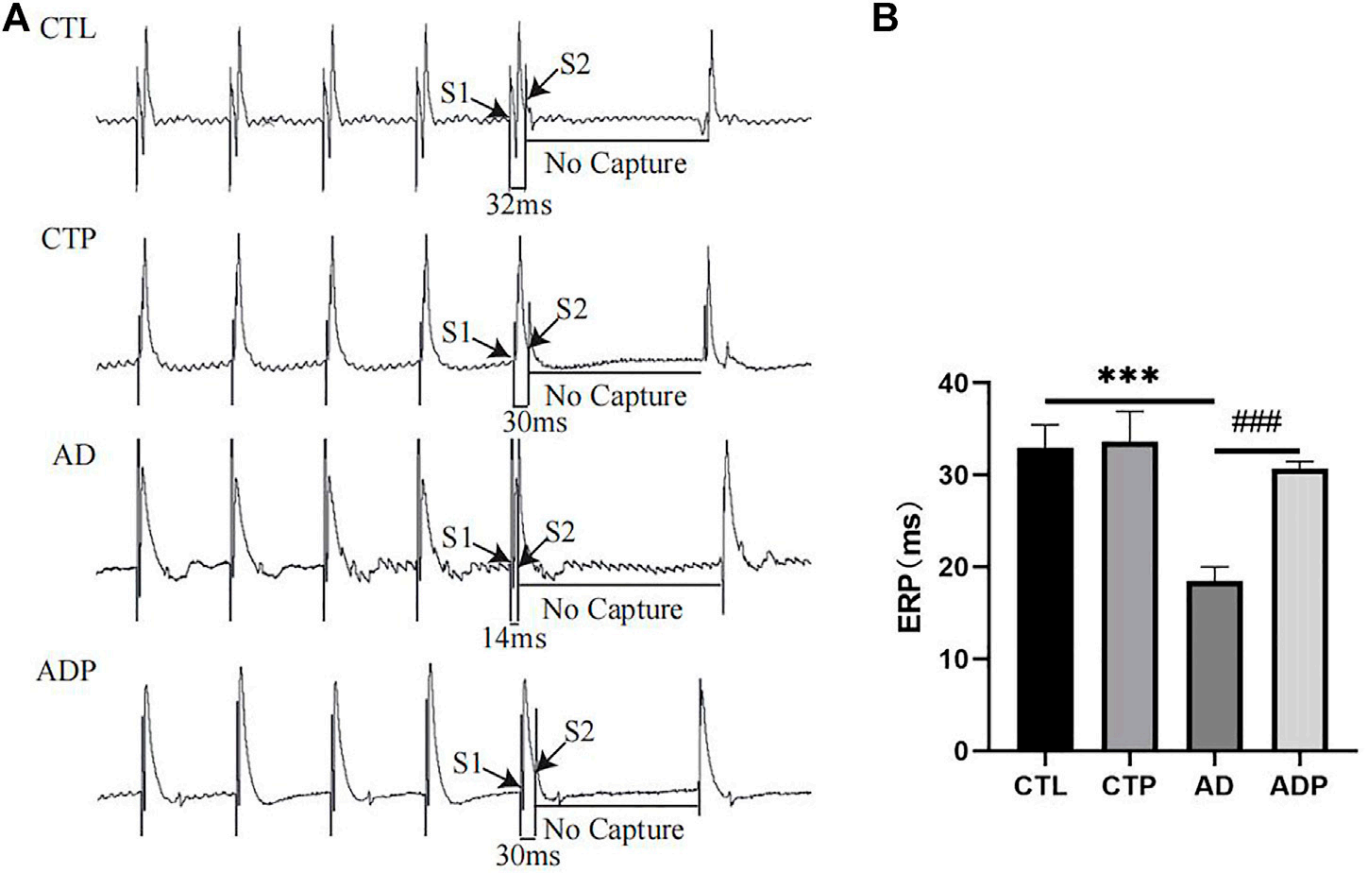
Effective refractory period. **(A)** Typical atrial ERPs at PCL of 200 ms. **(B)** Average ERP. *n* = 7 per group. *p < 0.05, **p < 0.01, ***p < 0.001 vs. CTL; #*p* < 0.05, ##*p* < 0.01, ###*p* < 0.001 vs. AD.

Representative APDs at PCL of 200 ms of the four groups were exhibited in Figure 3A. A significant prolongation of APD_50_ and APD_90_ was exhibited in the AD rats compared with the CTL rats, and the prolongation was prevented by pinocembrin in ADP rats (Figures 3B,C). Figure 4A showed the representative ERPs recorded from the four groups. The average ERP of the AD rats was remarkably shortened *versus* that of the CTL rats, and pinocembrin reversed the change in ADP rats (Figure 4B). In addition, no significant difference was exhibited in both of the APD and ERP between the CTL rats and the CTP rats.

### 3.4 Pinocembrin reduced atrial fibrillation susceptibility in anxiety disorder rats

**FIGURE 5:**
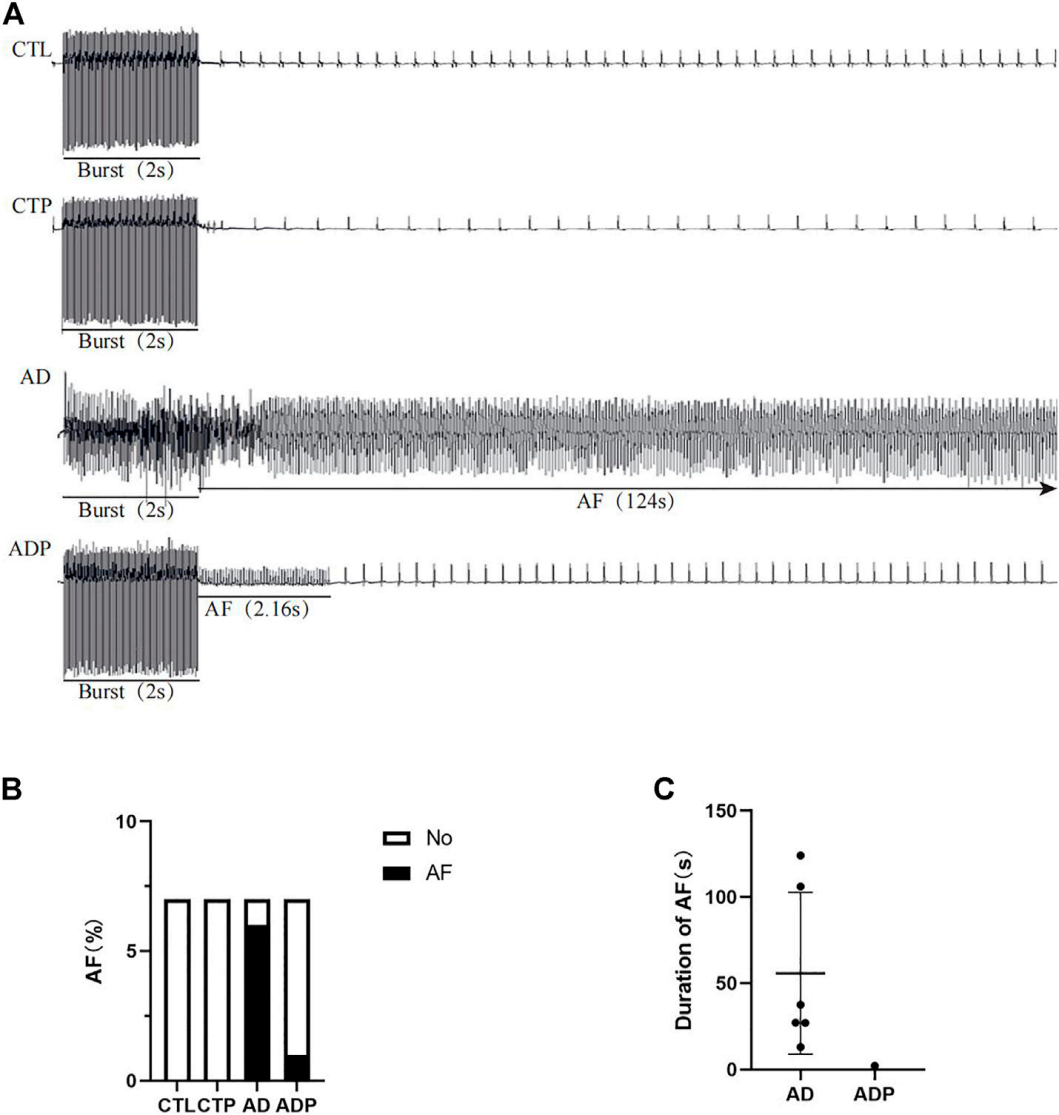
AF susceptibility. **(A)** Typical recordings of AF. **(B,C)** Inducibility and duration of AFs. n = 7 per group.

As shown in Figure 5A, the burst pacing procedure was conducted to induce AF in the four groups, and AF was only induced in the AD rats and ADP rats. The inductivity of AF in the AD rats was 85.71% (6/7), which was decreased by treatment with pinocembrin in the ADP group (14.29%, 1/7, Figure 5B). The average duration of AF in the AD group was 55.81 ± 46.85s, and AF lasted only 2.16s in the only ADP rat in which AF was successfully induced (Figure 5C).

### 3.5 Pinocembrin ameliorated the expression of ion channels and gap junction channel in anxiety disorder rats

**FIGURE 6:**
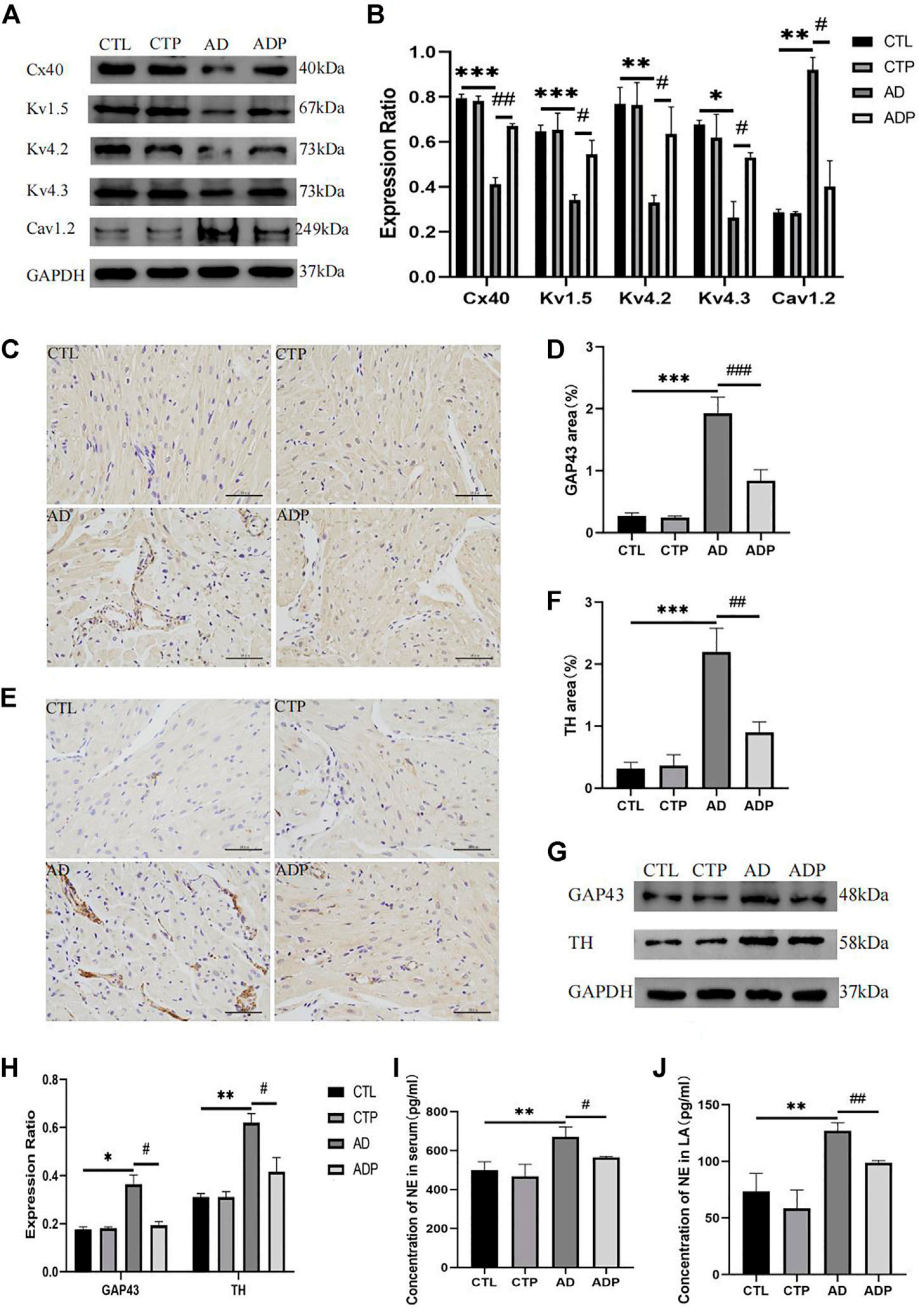
Ion channels remodeling, Cx40 expression, and autonomic remodeling. **(A,B)** Immunoblotting and quantitative analysis of Cx40, Kv1.5, Kv4.2, Kv4.3 and Cav1.2, *n* = 3 per group **(C ,D)** Typical images of GAP43 immunohistochemical staining and percentage of the GAP43 area, *n* = 5 per group. **(E,F)** Typical images of TH immunohistochemical staining and percentage of the TH area, n = 5 per group. **(G,H)** Immunoblotting and quantitative analysis of GAP43 and TH, *n* = 3 per grou4.3p. **(I,J)** Concentration of NE in serum and LA, *n* = 5 per group. *p < 0.05, **p < 0.01, ***p < 0.001 vs. CTL; #*p* < 0.05, ##*p* < 0.01, ###*p* < 0.001 vs. AD.

To further investigate the mechanisms of atrial electrophysiological abnormalities in AD rats, the content of atrial ion channels was assessed, including Kv1.5, Kv4.2, Kv4.3 and Cav1.2. The expression of Kv1.5, Kv4.2 and Kv4.3 was remarkably diminished, whereas the expression of Cav1.2 was increased in the AD rats *versus* the CTL rats (Figures 6A,B). In addition, the gap junction channel Cx40 was lower expressed in the AD rats compared with the CTL rats (Figures 6A,B). However, treatment with pinocembrin reversed the abnormal content of ion channels and Cx40 in the ADP rats, and there was no apparent difference between the CTL rats and the CTP rats.

### 3.6 Pinocembrin suppressed atrial autonomic remodeling in anxiety disorder rats

Figures 6C,E presented the typical TH and GAP43 immunohistochemical staining of the four groups. The positive stained area percentage of TH and GAP43 in the AD rats was remarkably expanded compared with the CTL rats, whereas pinocembrin apparently reduced the positive stained area percentage in the ADP rats (Figure 6D, F). The western bolt analysis of TH and GAP43 were exhibited in Figure 6G, which were consistent with the TH and GAP43 immunohistochemical staining. The content of TH and GAP43 was apparently increased in the AD rats compared with the CTL rats, but the content was attenuated by pinocembrin in the ADP rats (Figure 6H). Furthermore, a remarkable increase of NE concentration in serum and left atrium was exhibited in the AD rats, but the increase was suppressed by pinocembrin (Figures 6I, J). In addition, there was no apparent difference between the CTL group and the CTP group.

### 3.7 Pinocembrin reduced the content of TGF-β1, COL1 (collagen I), COL (collagen III) and attenuated atrial fibrosis in anxiety disorder rats

**FIGURE 7:**
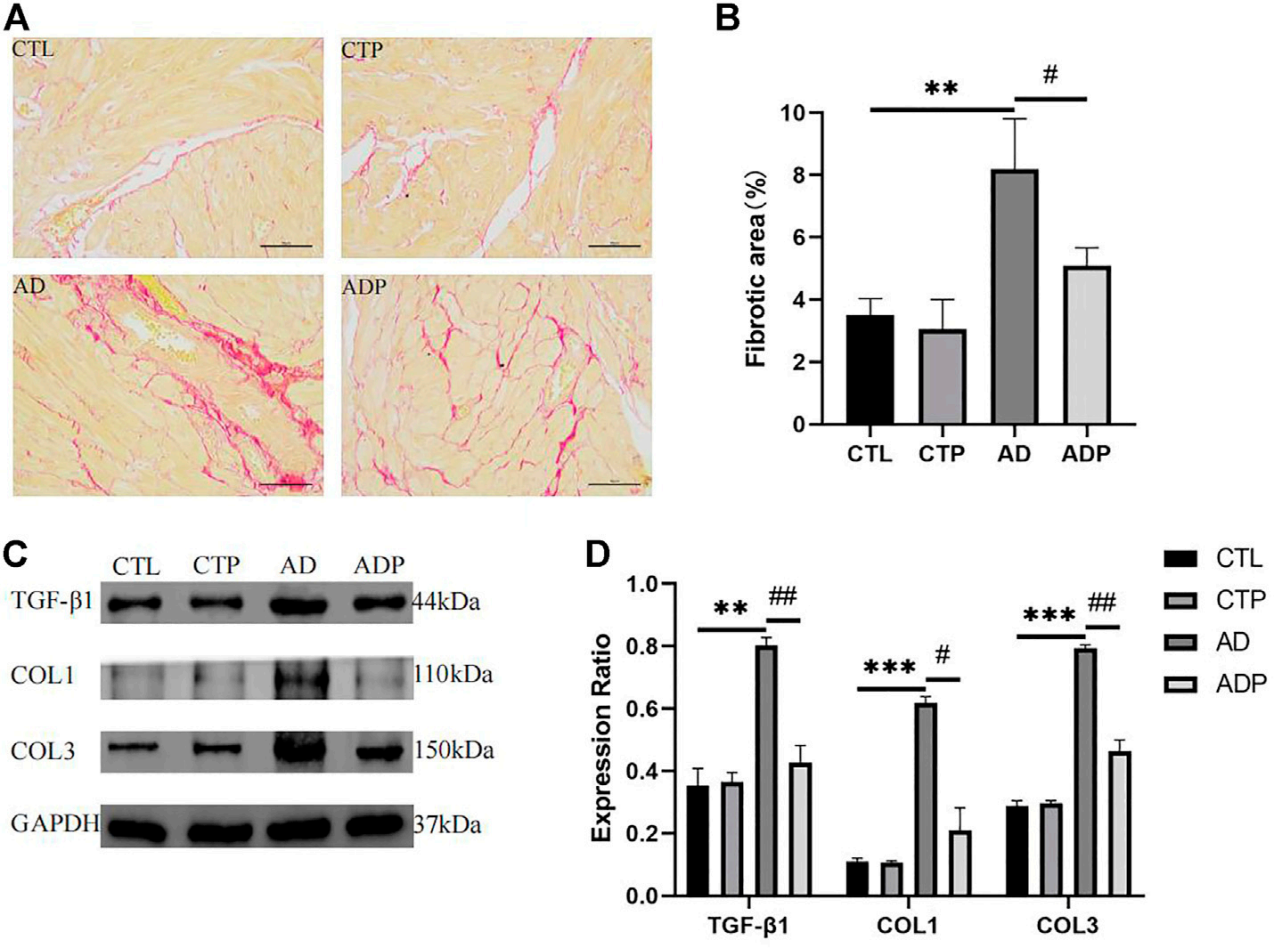
Atrial fibrosis. **(A,B)** Typical images of Sirius red staining and percentage of the fibrosis area, *n* = 5 per group. **(C,D)** Immunoblotting and quantitative analysis of TGF-β1, collagen I, and collagen III, *n* = 3 per group. *p < 0.05, **p < 0.01, ***p < 0.001 vs. CTL; #*p* < 0.05, ##*p* < 0.01, ###*p* < 0.001 vs. AD.

As shown in Figure 7A remarkably increased atrial fibrosis was exhibited in the AD rats compared with the CTL rats, whereas the atrial fibrosis was obviously diminished in the ADP rats treated with pinocembrin. Furthermore, TGF-β1, collagen I and collagen III were highly expressed in the AD rats, while the content of the three fibrosis related proteins was lowered by pinocembrin in the ADP rats (Figures 7C,D), indicating that pinocembrin could attenuate atrial fibrosis in the AD rats.

### 3.8 Pinocembrin alleviated oxidative stress through the nrf2/HO-1 pathway in anxiety disorder rats

**FIGURE 8:**
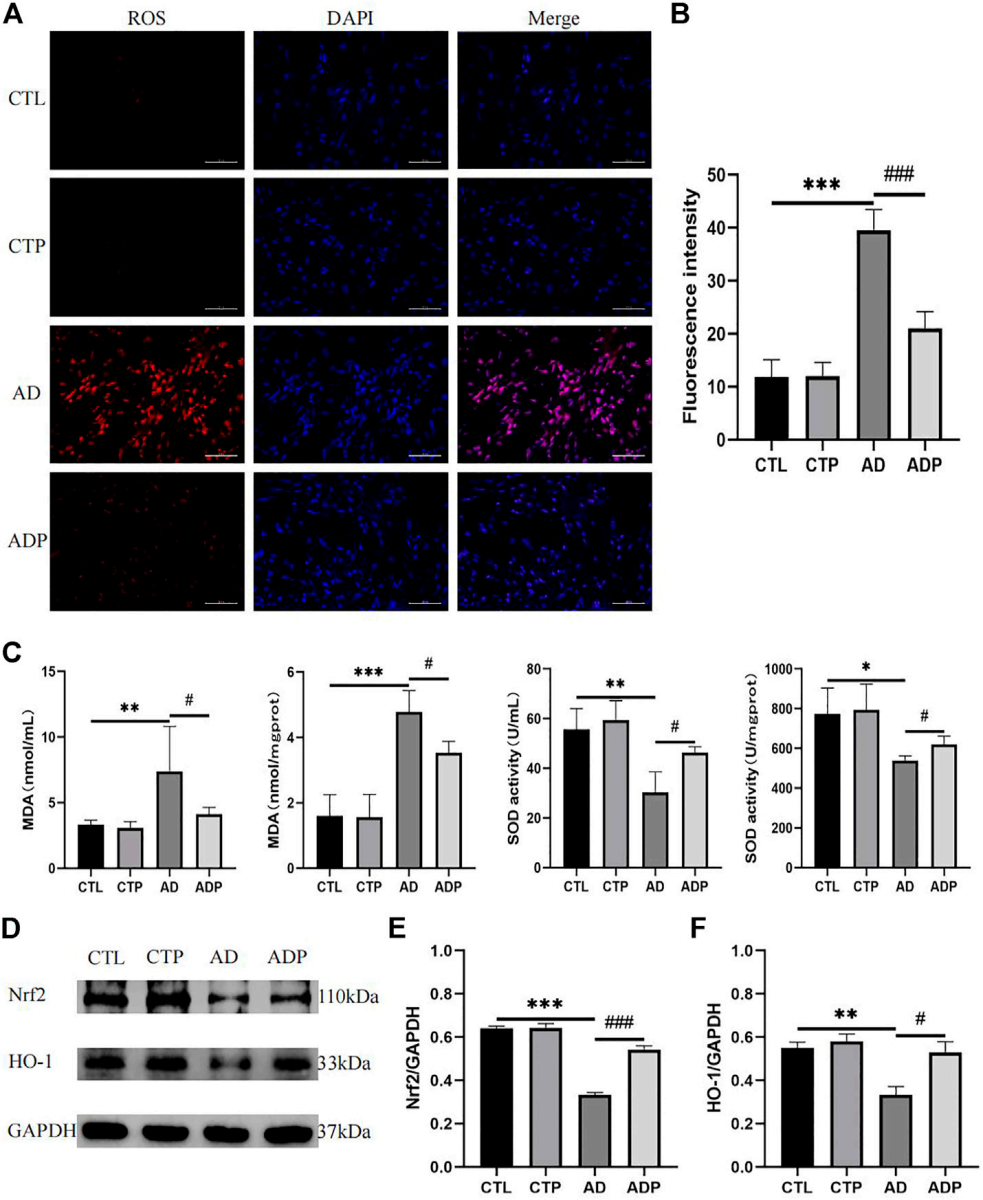
Oxidative stress *in vivo*. **(A,B)** Typical images and relative fluorescence intensity of ROS in LA, *n* = 5 per group. **(C)** The concentration of MDA and activity of SOD in serum and LA, *n* = 5 per group. **(D–F)** Immunoblotting and quantitative analysis of Nrf2, HO-1, *n* = 3 per group.

To explore the efficacy of pinocembrin on oxidative stress, the activity of SOD and concentration of MDA in serum and left atrium, and the level of ROS in atrium were detected. Compared with the CTL rats, the fluorescence intensity of ROS and the concentration of MDA were remarkably increased, but the activity of SOD was suppressed in the AD rats (Figures 8A–C). However, these abnormal changes were recovered by pinocembrin in the ADP group.

To further investigate the potential mechanism of pinocembrin on oxidative stress, we explored the Nrf2/HO-1 pathway. As shown in Figure 8D, the content of Nrf2 and HO-1 was lower in the AD rats than the CTL rats, illustrating that the Nrf2/HO-1 pathway was inhibited in the AD rats. However, the Nrf2/HO-1 pathway was upregulated by pinocembrin in the ADP rats with the improved level of Nrf2 and HO-1.

### 3.9 Pinocembrin alleviated H2O2-induced oxidative stress in HL-1 cells by the Nrf2/HO1 pathway *in vitro*

**FIGURE 9:**
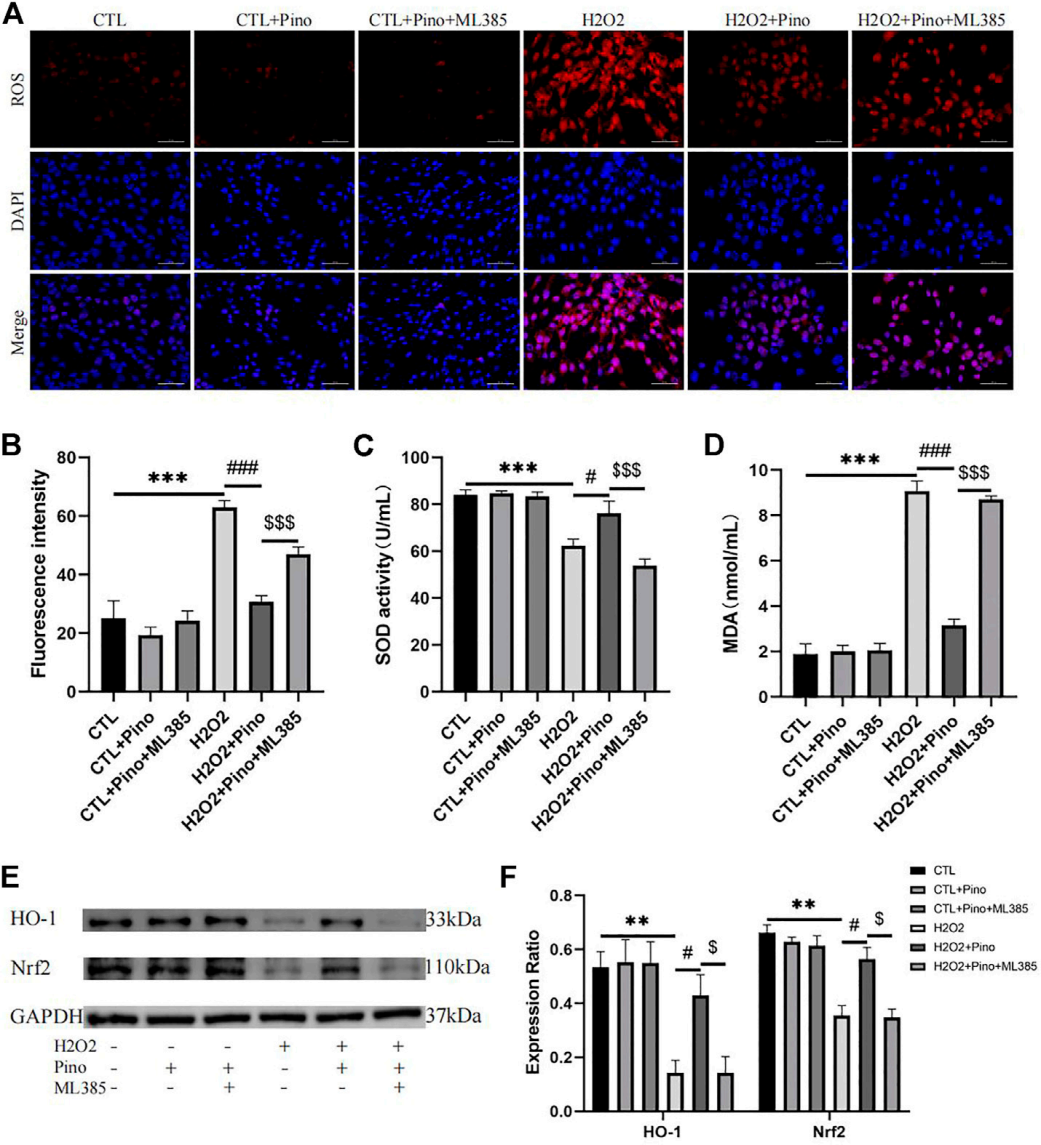
Oxidative stress *in vitro*. **(A,B)** Typical images and relative fluorescence intensity of ROS in HL-1 cells, *n* = 5 per group. **(C,D)** The activity of SOD and concentration of MDA in culture supernate, *n* = 5 per group. **(E,F)** Immunoblotting and quantitative analysis of Nrf2, HO-1, *n* = 3 per group.

To further demonstrate the antioxidant effect of pinocembrin is dependent on the Nrf2/HO-1 pathway, HL-1 atrial myocytes were cultured as previously reported and treated with H2O2 and Nrf2 inhibitor ML385 *in vitro*. As shown in Figures 9A–D, after 3 h of H2O2 treatment, ROS accumulation of HL-1 cells and MDA concentration in culture supernate were increased remarkably in the H2O2 group compared with the CTL group, while SOD activity in supernate was decreased significantly. However, ROS accumulation induced by H2O2 was significantly eliminated by the pretreatment of pinocembrin, MDA concentration in the supernate was apparently attenuated, and SOD activity was increased in the H2O2+Pino group. Significantly, these improvements were inhibited by the Nrf2 inhibitor ML385. Correspondingly, in the western blot analysis, pinocembrin improved the significantly decreased content of Nrf2 and HO-1 induced by H2O2, whereas these effects was abrogated by ML385. Therefore, the antioxidant stress effects of pinocembrin were at least partially realized *via* the Nrf2/HO-1 pathway.

## 4 Discussion

**FIGURE 10:**
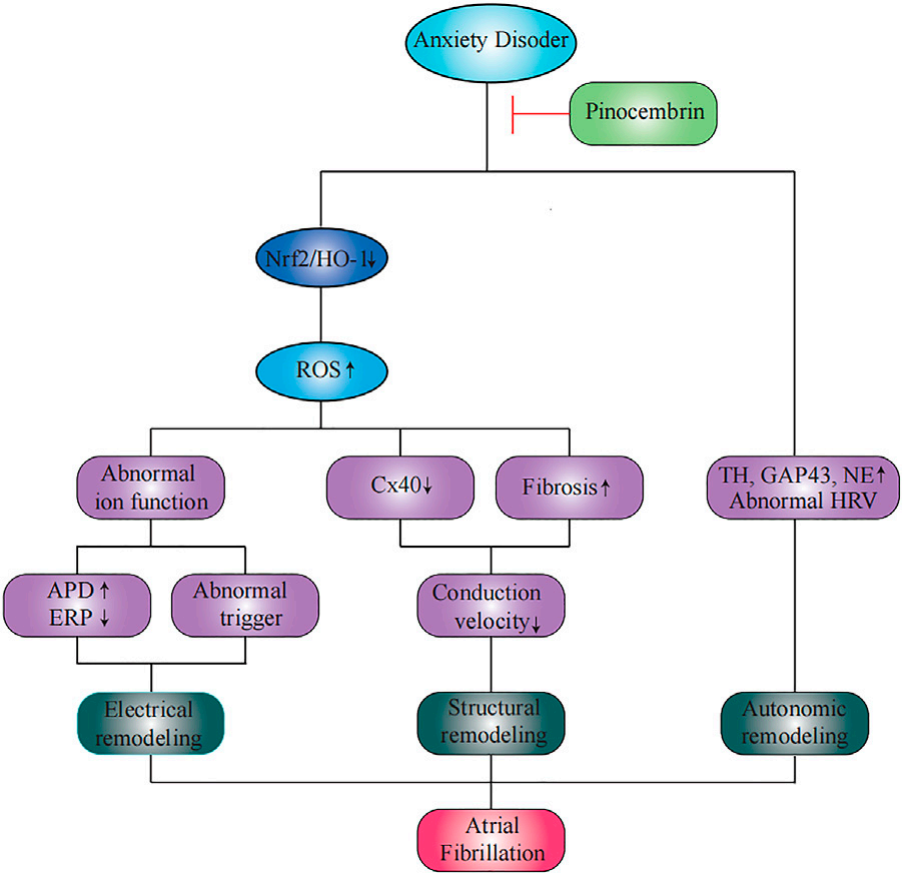
A summary of the effects of pinocembrin on atrial fibrillation susceptibility in rats with anxiety disorder.

In the current study, we demonstrated the efficacy of pinocembrin in reducing the AF susceptibility in anxiety disorder rats. Pinocembrin treatment improved anxiety-like behaviors and decreased atrial autonomic remodeling in anxiety disorder rats. Moreover, pinocembrin improved atrial electrical remodeling in anxiety disorder rats, including shortening of APD, prolongation of ERP, and restoring of ion channels related to action potential repolarization. In addition, pinocembrin improved atrial structural remodeling in anxiety disorder rats, including increased the expression of Cx40 and reduced atrial fibrosis. The improvement of pinocembrin on atrial electrical remodeling and structural remodeling in anxiety disorder rats may be attributed to its antioxidant effect through the Nrf2/HO-1 pathway. Pinocembrin ultimately reduced the AF susceptibility through the above effects in anxiety disorder rats (Figure 10).

### 4.1 Pinocembrin and atrial electrical remodeling

Anxiety disorder and AF often co-occur in patients. Studies with small samples have shown that AD occurred in approximately one-third of patients with AF (Patel et al., 2013; Polikandrioti et al., 2018). A meta-analysis exhibited that AD has made a significant contribution to cardiac events, including an increased risk of cardiovascular mortality, coronary heart disease, and heart failure (Emdin et al., 2016). Patients with AD have been identified to be characterized by prolonged atrial electromechanical delay detected by tissue Doppler echocardiography (Oksuz et al., 2017), which was a herald of AF.

To induce and maintain AF, triggers and substrates are required. Triggers include early afterdepolarization (EAD) and delayed afterdepolarization (DAD), which could induce arrhythmias in the presence of appropriated substrates. Prolonged APD could evoke EAD (Killeen et al., 2007), and intracellular calcium overload could elicit both EAD and DAD (Ohta et al., 1987; Chang et al., 2018). Substrates include uneven prolongation of APD, reduction of conduction velocity, shortening of ERP, and widening of the critical interval of re-excitation (APD-ERP) (Tse et al., 2021). The current study exhibited prolonged APD and shortened ERP in the AD rats, which promoted the occurrence and development of AF, whereas pinocembrin could recover these abnormalities.

The transient outward potassium current (I_to_) and the inward calcium current (I_Ca-L_), generated by Kv4.2, Kv4.3 and Cav1.2, respectively, are the two main currents which contribute to the cardiac repolarization in rats. I_to_ contributes to the early rapid repolarization and the initial part of the platform (Greenstein et al., 2000), and reduced I_to_ results in prolonged APD (Greenstein et al., 2000). In contrast, genetically increased K^+^ current in cardiomyocytes could shorten APD, suppress susceptibility to EAD, and prolong ERP (Nuss et al., 1999). I_Ca-L_ is the main current in the plateau, and increasd I_Ca-L_ prolongs the plateau phase, thus prolonging APD (Anderson, 2004). Sustained activation of I_Ca-L_ leads to pathological myocardial remodeling and imbalance of calcium homeostasis, which are closely linked to the arrhythmic phenotype (Zobel et al., 2007). Ultra-rapidly activating (I_Kur_) is generated by potassium current through the Kv1.5 channel, and decreased function of which is associated with AF (Olson et al., 2006; Yang et al., 2010). Our study indicated a significant suppress of Kv1.5, Kv4.2, Kv4.3 and increase of Cav1.2 in the AD rats, which could be reversed by pinocembrin.

### 4.2 Pinocembrin and atrial autonomic remodeling

Previous study has demonstrated that anxiety disorder can lead to the increased sympathetic function and catecholamine overload, which are important triggers of atrial fibrillation (Severino et al., 2019). In the current study, the positive expression in the TH and GAP43 immunohistochemical staining was increased in AD rats, along with the upregulation of the content of TH and GAP43, indicating that the activity of the cardiac sympathetic nerve was enhanced. HRV is used to assess autonomic nervous function and can be affected by mental factors (Shu et al., 2018). SDRR, RMSSD and HF reflect the parasympathetic activity, while LF reflect the sympathetic activity (Author Anonymous, 1996). In patients with anxiety disorder, RMSSD was suppressed and the LF/HF ratio was enhanced (Christou-Champi et al., 2015). In our study, the AD rats exhibited a reduction of average RR, SDRR, RMSSD, and HF, but an increase of LF and LF/HF, illustrating that the sympathetic nervous system was activated and the parasympathetic nervous system was inhibited. In addition, the serum NE was increased in AD rats. Catecholamine overload can lead to the increased extracellular matrix and abnormal calcium handle, which increases the risk of AF (Boluyt et al., 1995; Nef et al., 2010). However, the imbalance of autonomic nervous function and increased serum NE in AD rats was reversed pinocembrin, demonstrating that pinocembrin suppressed AF susceptibility at least in part by modulating atrial autonomic remodeling.

### 4.3 Pinocembrin and atrial structural remodeling

Atrial fibrosis is a potential substrate of AF. On one hand, fibrosis forms a conduction barrier to block local electrical conduction, on the other hand, fibroblasts interact with cardiomyocytes, promote re-entry and spontaneous ectopic activity (Andrade et al., 2014). Previous study has demonstrated that connexins play a vital role in AF by promoting the conduction of action potential between cardiomyocytes (Chaldoupi et al., 2009). Cx40 is a major connexin in the atria, and cx40 knockout mice exhibited an increased AF susceptibility (Kirchhoff et al., 1998). In our present study, increased atrial fibrosis was found in the AD rats, and the content of TGF-β1, Collagen I and Collagen III was also upregulated, but the expression of cx40 was decreased in the AD rats. However, the increased atrial fibrosis and decreased cx40 were recovered by pinocembrin, indicating that pinocembrin can ameliorate atrial structural remodeling in the AD rats.

### 4.4 Pinocembrin and oxidative stress

Oxidative stress refers to the imbalance between pro-oxidants and antioxidants (Lichtenberg and Pinchuk, 2015). Previous study has illustrated that oxidative stress was increased in anxiety disorder, and antioxidants can ameliorate neurological damage caused by psychiatric disorders (Jin et al., 2014). Our present study revealed that the generation of ROS and the concentration of MDA were increased, but the activity of SOD was reduced in the AD rats, indicating that oxidative stress was intensified in the AD rats. However, those abnormalities were recovered by pinocembrin in the ADP rats.

Oxidative stress can affect the atrial electrical remodeling and thus increase the AF susceptibility. H2O2 can lead to the overload of Na^+^ and Ca^2+^ in cardiomyocytes, prolong cardiac action potential and increase early after depolarizations (EADs) (Sovari, 2016). In addition, oxidative stress can reduce the K^+^ currents, which can be recovered by SOD and NADPH oxidase (NOX) inhibitor apocynin (Shimoni et al., 2005). Moreover, ROS can activate calmodulin-dependent protein kinase II (CaMKII), which leads to the abnormal Ca^2+^ handling in cardiomyocytes and increases intracellular Ca^2+^ (Mesubi and Anderson, 2016).

Furthermore, oxidative stress contributes to the atrial structural remodeling and thus provides substrate for the development of AF. Oxidative stress promotes the progression of cardiac fibrosis. NOX_2_ and NOX_4_ are major sources of ROS production in the heart. Activation of NOX_2_ and its downstream JNK/NFAT pathway can promote cardiac fibroblast generation (Fujii et al., 2005), while NOX_4_ participates in TGF-β-induced cardiac fibroblasts differentiation by activating smad2/3 pathway (Cucoranu et al., 2005). And ROS scavengers (NAC) significantly reduced fibrosis in angiotensin II treated cardiomyocytes by inhibiting TGF-β1/SMADs signaling pathway (Zhu et al., 2020). In addition, oxidative stress can reduce the expression of cx43 in the heart, slowing the conduction velocity and increasing the electrical heterogeneity, which can be reversed by antioxidants (Sovari et al., 2013).

Nrf2/HO-1 is an antioxidant pathway that exhibits endogenous protective effects against damages induced by ROS. In response to stress, Nrf2 separates from its inhibitor Keap1 and transfers into nucleus, promoting the production of antioxidant enzymes represented by HO-1, which could remove heme, produce biliverdin, iron ion and carbon monoxide (Loboda et al., 2016). Previous studies have demonstrated that Nrf2/HO-1 performed protective effects on the nervous system and cardiovascular system. As to Alzheimer’s disease and Parkinson’s disease, oxidative stress exacerbated the progression of which, while inhibition of ROS and promotion of the Nrf2/HO-1 pathway can alleviate the neuronal apoptosis and improve the symptoms (Feng et al., 2016; Li et al., 2018). Nrf2/HO-1 pathway can suppress myocardial apoptosis and reduce the incidence of ventricular arrhythmia induced by myocardial ischemia-reperfusion (Enayati et al., 2018). Our previous study demonstrated that pinocembrin can improve the cardiac function and reduce myocardial apoptosis and cardiac fibrosis in rats with heart failure by promoting Nrf2/HO-1 pathway (Chen et al., 2021). Our study exhibited a remarkable inhibition of the content of Nrf2 and HO-1, but pinocembrin administration improved the Nrf2/HO-1 pathway in the ADP rats. In addition, in H_2_O_2_-treated HL-1 cells, pinocembrin promoted the Nrf2/HO-1 pathway, but this promotion was eliminated by ML385, a Nrf2 inhibitor, suggesting that Nrf2 is the therapeutic target of pinocembrin exerting the antioxidant effects.

## 5 Conclusion

Pinocembrin can reduce AF susceptibility in anxiety disorder rats, with the inhibition of electrical remodeling, autonomic remodeling, structural remodeling, and oxidative stress. Therefore, pinocembrin is a promising treatment for anxiety disorder patients with AF.

## 6 Limitations

Although the animal study objectively demonstrate pinocembrin is a potentially promising pharmacological candidate, additional studies and clinical trials are required to determine its specific intracellular sites of action and derivative targets in order to fully understand the mechanism of pinocembrin and to further validate its clinical medical applications.

## Data Availability

The original contributions presented in the study are included in the article material, further inquiries can be directed to the corresponding authors.
